# “It’s Just a Band-Aid on Something No One Really Wants to See or Acknowledge”: A Photovoice Study with Transitional Aged Youth Experiencing Homelessness to Examine the Roots of San Diego’s 2016–2018 Hepatitis A Outbreak

**DOI:** 10.3390/ijerph17134721

**Published:** 2020-06-30

**Authors:** Jennifer K. Felner, Talia Kieu, Andrew Stieber, Hunter Call, Daniel Kirkland, Amanda Farr, Jerel P. Calzo

**Affiliations:** 1 School of Public Health, San Diego State University, San Diego, CA 92123, USA; tkieu-w@sdsu.edu (T.K.); andrewcstieber@gmail.com (A.S.); jcalzo@sdsu.edu (J.P.C.); 2 Institute for Behavioral and Community Health, San Diego State University, San Diego, CA 92123, USA; 3 Independent Researcher, San Diego, CA 92123, USA; huntercall29@gmail.com (H.C.); kirklanddaniel6@gmail.com (D.K.); 4 Child and Adolescent Services Research Center, School of Medicine, University of San Diego, San Diego, CA 92123, USA; afarr@health.ucsd.edu

**Keywords:** photovoice, community-based participatory research, hepatitis A virus, homelessness, youth, stigma, qualitative methods

## Abstract

San Diego, California is consistently ranked among regions with the highest rates of homelessness in the United States. From 2016 to 2018, San Diego experienced an unprecedented outbreak of hepatitis A virus (HAV), largely attributed in media and public health discourse to the region’s growing population of people experiencing homelessness. Little attention, however, was devoted to examining the experiences and needs of this population, particularly transitional aged youth (TAY, aged 18–24) experiencing homelessness who may have been uniquely affected by the outbreak. This community-based participatory research study leveraged diverse qualitative methods, principally photovoice, to explore how the social and built environment shapes health among TAY experiencing homelessness in San Diego, how these environments may have contributed to the HAV outbreak, and TAY’s perceptions of HAV-related public health interventions. Emergent findings include stigmatization of TAY and other people experiencing homelessness, interventions that failed to address root causes of the outbreak, and interactions with housing-related and other social support resources that limit rather than support economic and social mobility. Findings have implications for understanding how media and public discourse, public health interventions, and availability and delivery of resources can contribute to and perpetuate stigma and health inequities faced by TAY experiencing homelessness.

## 1. Introduction

In preventing and mitigating the harms of infectious diseases that disproportionately affect marginalized populations, consideration of social determinants of health—such as income, social and built environments, and access to healthcare—can help ensure that public health efforts do not perpetuate or expand health inequities. The case of recent hepatitis A virus (HAV) outbreaks in the United States (U.S.), characterized in many urban settings as an issue primarily affecting people experiencing homelessness (PEH), illustrates the need for researchers, public health authorities, and policymakers to consider the social determinants of infectious disease outbreaks and related implications for intervention rather than narrowly focusing on containment of disease transmission.

HAV is a preventable communicable disease affecting the liver, spread primarily through foodborne transmission or close physical contact with someone who is infected (i.e., fecal-oral route) [1]. In the past decade, cases of HAV have remained relatively low across the U.S., largely due to increases in vaccinations among infants [2]. Beginning in late 2016, however, new person-to-person HAV outbreaks emerged in multiple countries that previously had low incidence rates, including the U.S., where outbreaks predominantly affected people reporting illicit drug use, PEH, and other “high risk” groups [3,4]. In March 2017, San Diego, California was among the first regions in the U.S. to declare a public health emergency related to the new outbreaks, with the earliest known cases traced to November 2016 [4,5]. With acute awareness of the infectious disease epidemiology of HAV [6,7], the Health and Human Services Agency of the County of San Diego and its partners utilized a variety of intervention methods to mitigate the outbreak. These efforts included broad public health messaging about HAV transmission and prevention (e.g., news reports and signage), and an emphasis on preventive public health interventions for high risk groups, including PEH. Such interventions included mobile vaccination clinics, installation of temporary public handwashing stations, sanitation of sidewalks and public spaces, and distribution of personal hygiene kits [5,8]. By April 2018, the number of cases in San Diego significantly decreased, and the HAV emergency status was lifted. At the time of drafting this manuscript, 27 states in the U.S. have active HAV outbreaks, with more than 20,000 HAV-related hospitalizations and 300 deaths nationwide [4].

Although efforts to address the HAV outbreak in San Diego focused on informing the public about HAV risk and prevention, and intervening with populations at elevated risk, a predominant focus of the public health, policy, and media narrative concerning the outbreak was the connection between HAV and homelessness [3,9]. Indeed, more than 700 outbreak-related cases and 21 outbreak-related deaths have been reported in San Diego to date [4], with nearly half of all HAV cases in San Diego (49%) among PEH [10]. In addition, PEH were more likely to be hospitalized and to die from HAV-related complications than those who were stably housed [10]. These disparities in HAV rates between the housed and unhoused have been attributed to PEH’s limited access to hygiene and sanitation resources (e.g., public bathrooms) in San Diego [5,11]. However, theoretical frameworks suggest that disparities in HAV by housing status may also be tied to multi-level marginalization and stigmatization of PEH [12,13,14,15,16].

Stigma can be enacted in multiple ways, including attitudinally, such as the perception and treatment of PEH as “outsiders” within their own communities and neighborhoods, and systemically through exclusion and isolation of people who lack stable housing from social support resources, employment opportunities, and engagement in healthcare and health-supporting systems [17,18]. Because many of the public health interventions addressing the HAV outbreak in San Diego were temporary (e.g., handwashing stations, power-washing streets), yet the theoretical social determinants of the catastrophic outbreak were more upstream and chronic (i.e., societal stigmatization of PEH), a closer analysis of understudied social forces undergirding the HAV outbreak was warranted. This study examines, from the perspective of a specific group of PEH (transitional aged youth [“TAY”, aged 18–24]), how and in what ways stigma contributed to the HAV outbreak in San Diego, and (through the lens of the HAV outbreak) how stigma is enacted and perpetuated in policies and resources that affect the lives of PEH.

### Studying the Outbreak from the Perspective of Transitional Aged Youth Experiencing Homelessness (TAY)

At the time of the HAV outbreak, the study “academic co-leads” (J.K.F. and J.P.C.) observed that the voices of PEH were largely absent in related news media and communications from public health officials, despite receiving the bulk of the blame for its spread. To better understand if this observation was accurate, they engaged in conversations with leaders and staff at a local community-based organization (with whom they had an established public health practice relationship) providing temporary emergency shelter and long-term housing to TAY experiencing homelessness in the San Diego region. The leaders and staff shared the concern that PEH—in particular, TAY—were excluded from media and public health discourse about the outbreak, and the planning and implementation of the public health response. This absence was particularly glaring given that, as a state, California has the highest population of homelessness in the country and, collectively, the City and County of San Diego have the fourth largest population of PEH among U.S. cities and regions [19]. Furthermore, a sizable proportion of PEH in San Diego [20] and nationally [21,22] are TAY, whose intersecting identities as young and unhoused may situate them in a unique social location with respect to the outbreak and associated public health interventions [23].

While there are various members of the community of PEH in San Diego (e.g., TAY and other adults experiencing homelessness, service providers), based on initial engagement and feedback from the community-based organization and their collective expertise in youth development research, the academic co-leads sought to center the experiences, needs, and ideas of TAY experiencing homelessness with respect to the HAV outbreak. With support from the community-based organization, the academic co-leads recruited four TAY who were participating in local housing-related resources to join them as “community co-leads” of a community-based participatory research (CBPR) study to broadly examine how TAY experiencing homelessness perceived and experienced the HAV outbreak and related interventions.

CBPR is an orientation to research that eschews traditional academic-driven science in service of involving community members authentically in all stages of research from issue selection to findings dissemination. CBPR processes are meant to democratize the production of knowledge such that community members’ perspectives, experiences, needs, and ideas are prioritized and sustainable community health improvements may be realized [24,25,26]. A key strength of CBPR is the inclusion of both emic (insider/community) and etic (outsider/academic) perspectives in research on health-related phenomena that either group may miss or take for granted from their unique vantage points [27]. In addition, CBPR is strengthened by its synthesis of community partners’ lived-experience and nuanced understanding of and investment in community assets and needs with academic partners’ skills in study design, data collection, and analysis, and understanding of public health systems [28,29]. Together, these emic-etic perspectives aim to produce knowledge directly relevant for culturally grounded public health planning and policy development.

Although CBPR approaches to studying public health phenomena are increasingly common, including CBPR studies of health needs and assets among PEH (e.g., [30,31,32]), the present study represents a novel application of CBPR in the context of the 2016–2018 HAV outbreak in San Diego from the perspective of TAY experiencing homelessness, given that past research on the outbreak (and outbreaks across the U.S.) have focused more on documenting epidemiologic patterns in associated morbidity and mortality rather than on the actual lived-experiences of those most affected. The study team ultimately elected to use qualitative methods, principally photovoice, to examine how the social and built environment shapes health among the community of TAY experiencing homelessness, how these environments may have contributed to the HAV outbreak, and perceptions of HAV-related public health interventions among TAY experiencing homelessness (hereafter “TAY”). In addition, while the broad goal of the study was pre-identified by the academic co-leads with support from the community-based organization, the specific research questions and methods were intentionally undetermined so that the academic co-leads and community co-leads could work collaboratively to design and carry out the study.

## 2. Materials and Methods

From July 2018 to May 2019 the “Action4Health” study team—consisting of the academic co-leads, the community co-leads (including H.C. and D.K.), and eight student research assistants (including T.K., A.S., and A.F.)—met approximately bi-weekly to identify the study’s research questions and methods, collect and analyze data, and plan for findings dissemination. The community co-leads provided written informed consent to participate in the study and were compensated $30 per research meeting attended and $40 per presentation/session facilitated (i.e., member-checking sessions, community forum). Over the course of the study, two of the community co-leads voluntarily disengaged from the study.

Over the course of the study, team members engaged in an iterative process of research question identification, methods selection, data collection and analysis, and member-checking, with each step in the research process informing the next. Table 1 provides an overview of the primary and secondary methods, study validation techniques, the timing and goal of each method/validation technique, samples, and study team members involved in data collection. The following sections outline the identification of research questions and methods, and data collection and analysis.

### 2.1. Research Question Identification

The academic co-leads guided the study team in multiple group-level discussions and exercises to finalize the study goals and identify specific research questions. The community co-leads brought to these discussions novel insights about their experiences with homelessness and perceptions of the HAV public health response in San Diego. For example, in an early study team meeting, one of the community co-leads discussed concerns about the motivations for vaccinating TAY and other PEH in the wake of the outbreak, and the relative safety and utility of the HAV vaccine: “Are they only doing this so the disease won’t spread to the high class? Do vaccinators actually care about the health of these people? And, what if the vaccination is [harmful]?”. The community co-leads acknowledged the widespread attention the outbreak was receiving in the media and societal blame placed on PEH, while also personally perceiving the outbreak as relatively removed from their own lives. They indicated that they did not know anyone who had contracted HAV and emphasized the need for policymakers to address more salient issues contributing to the health of TAY and other PEH rather than the outbreak itself (e.g., lack of jobs, opportunities for permanent housing for TAY). These early discussions allowed the community co-leads to guide the study team in an inquiry that would address not only the outbreak, but broader and more critical issues of relevance to TAY, including the stigmatization of homelessness. Based on these discussions and insights, the study team identified an overall goal of examining the social and environmental determinants of the HAV outbreak and health among TAY, and answering the following overarching research question: “How has the public health response to the HAV outbreak perpetuated the stigma of homelessness among TAY?”

As the study evolved, the community co-leads raised concerns about the ways in which societal stigma limits opportunities for well-being and securing stable housing. These discussions included phenomena documented in existing literature, such as the privatization of public spaces and associated criminalization of mundane activities (e.g., sitting or sleeping on a park bench) and the desire of housed residents in middle- and high-income neighborhoods to actively protest the presence of homeless shelters or PEH in their neighborhoods (i.e., “Not in My Back Yard”, or, NIMBYism) [14,33,34,35]. These discussions yielded two additional research questions to guide the study: “How do the physical, social, and logistical constraints of homelessness impact the health and well-being of TAY?” and “How can public health and social service systems cultivate resources for growth among TAY, not just maintenance?” i.e., what actions can be taken to ensure that TAY can thrive rather than simply survive?

### 2.2. Methods Selection

Based on the initial research question and identified goals, the study team selected photovoice as a primary methodology. Photovoice is an established CBPR method blending photography and/or videography, storytelling, and community-driven action for collective change [36]. Photovoice typically involves an iterative process in which community members or community-academic teams (1) capture people’s lived realities via photographs and/or videos, with data collection motivated by a question or specific prompt related to an overarching research question, and (2) engage in critical dialogue and analysis about the photos/videos.

A common approach to analyzing photovoice data is the “SHOWeD technique” which guides critical analysis and discussion of selected photos or videos [37,38]. SHOWeD involves individually responding in written (~1–3 sentences) or verbal form to the following questions regarding selected photos/videos: “(1) What do you **S**ee here? (2) What is really **H**appening here? How does this relate to **O**ur lives? **W**hy does this situation, concern, or strength **E**xist? and What can we **D**o about it?”. The written or verbal narratives are then used to guide group-level discussions towards the identification of policy-relevant recommendations for improving health. At the completion of a photovoice study, selected photos/videos and accompanying SHOWeD narratives are exhibited to an appropriate audience, generally including policymakers and other key stakeholders, to encourage dialogue about implications for community health improvements [36,38,39].

In addition to photovoice, the study team identified two supporting qualitative methods to be leveraged—historical analysis of news/media articles related to the HAV outbreak published between May 2017 and January 2019, and asset mapping of TAY-specific resources (e.g., TAY shelters) and interventions related to the HAV outbreak (e.g., handwashing stations) in San Diego. To assess the validity of emergent findings, the study team conducted stakeholder interviews with individuals working or volunteering with organizations serving PEH, individuals working for the City/County of San Diego, and public health officials, and held member-checking sessions with groups of TAY.

### 2.3. Data Collection

#### 2.3.1. Primary Method: Photovoice

After a literature review, the academic co-leads trained the study team in the logistics of photovoice and the SHOWeD technique, including ethical considerations when collecting visual data (e.g., excluding faces and other identifiable characteristics). Beginning in August 2018, members of the study team (including the academic and community co-leads) collected photographs and videos during multiple group outings in various locations in San Diego. These locations were identified by the community co-leads as places where they and other TAY engaged in social activities, accessed resources, or relaxed. In some instances, the study team collected data in response to specific prompts, some of which were elicited by supporting methods such as historical analysis, for example: “What kinds of housing and social support-related resources do TAY access?”, “What resources do TAY use to stay safe and clean?”, and “Walk the study team through ‘a day in the life’ of a TAY accessing specific resources or services, (e.g., public showers), or meeting basic needs (e.g., charging a cellphone)”. In other instances, the community co-leads guided the study team in the collection of photos/videos to broadly capture the social and built environment among TAY and other PEH without the use of specific prompts or questions. Over the course of the study, more than 250 photos and videos were collected from five neighborhoods/locations in San Diego. The study team periodically engaged in SHOWeD sessions (*n* = 12) of selected photos/videos, which then informed future data collection. Written/verbal SHOWeD narratives were saved for future analysis, and SHOWeD discussions were audio recorded and transcribed verbatim by the student research assistants.

Given that the study team included a small number of TAY, it was critical that more TAY perspectives be gathered to further contextualize emergent findings and guide additional data collection. In January 2019, the study team held a community-based SHOWeD session at a local drop-in center for TAY. TAY were eligible to participate in the session if they were 18 to 24-years-old and had a history of housing instability or homelessness. Participating TAY (*n* = 7) had an average age of 21.86 ± 1.57 (range: 19–24), were diverse in terms of their race/ethnicity, sexual orientation, and gender identity (*n* = 5 self-identified as people of color; *n* = 4 self-identified as members of lesbian, gay, bisexual, transgender, and/or queer [LGBTQ] community). The session involved members of the study team (including the community co-leads) presenting participating TAY with four photographs collected by the study team and asking them to write or dictate to a student research assistant their responses to the SHOWeD prompts for each photo. The study team then guided participants through group-level discussions about the photos and written narratives. The session lasted 90 minutes and was audio recorded and transcribed verbatim by the student research assistants. Participating TAY provided written informed consent and received $30 for their time.

#### 2.3.2. Supporting Methods: Historical Analysis and Asset Mapping

The supporting methods of historical analysis of news media and asset mapping occurred in tandem with the photovoice data collection and SHOWeD sessions (see Table 1). The historical analysis (*n* = 30 news articles) focused on understanding how the media portrayed the HAV outbreak and contextualizing the photovoice data. These data were collected by the academic co-leads and student research assistants prior to the formation of the full study team and continued with guidance from the community co-leads. The asset mapping process involved identifying and documenting the location of specific HAV interventions implemented by the County of San Diego (e.g., handwashing stations, vaccination clinics, porta-potties) and resources available to TAY (e.g., drop-in centers, public showers) to understand how HAV interventions and available resources were distributed geospatially across San Diego, and to further contextualize the photovoice data. The student research assistants gathered information on HAV interventions from the County of San Diego website and on resources for TAY from 2-1-1 San Diego, a resource hub for community-based and public services. Quantum GIS Software [40] visually depicted the location of each resource. The community co-leads provided guidance on additional resources to include on the asset map (de-identified map available upon request).

#### 2.3.3. Validation Techniques: Stakeholder Interviews and Member-Checking

Prior to the formation of the full study team, the academic co-leads met with relevant stakeholders to develop the broad scope of the project and to identify potential community partners. Once the study team was formed, additional periodic stakeholder interviews were held to contextualize the photovoice data and to examine the extent to which emergent findings resonated with those working directly with TAY and other PEH, and/or for the City/County of San Diego. Interviewees (*n* = 10) provided permission for study team members to write field notes during and after the interviews.

The study team also held member-checking sessions with two groups of TAY with a history of homelessness. Specifically, they met with members of a youth advisory board focused on improving services for youth experiencing homelessness in San Diego (*n* = 4) and with a group of TAY participating in a local housing program for LGBTQ youth (*n* = 6). Member-checking sessions focused on sharing key findings from the study, including supporting photos and SHOWeD narratives, to assess the extent to which findings reflected the experiences of TAY who were not involved with the data collection process and who have perspectives and experiences that may differ from members of the study team. Member-checking sessions also informed future data collection. TAY participating in member-checking provided permission for study team members to write field notes during and after the sessions.

### 2.4. Data Analysis

The study team identified themes across the data sources using iterative, participatory analysis processes [41,42,43]. Drawing on the outputs from the primary and secondary qualitative data sources, the team engaged in multiple analytic discussions to generate thematic categories that reflected the range of emergent constructs. For example, in one exercise the academic co-leads asked the members of the study team to reflect on one or more of the research questions and ask how different sources of data helped to answer that question, thereafter creating visual maps of relationships between emergent themes and subthemes. These exercises continued until study team members reached agreement about the final thematic categories supported by the multiple sources of data.

A critical component of CBPR involves translating knowledge produced for practice and policy-related changes to bridge the research and action gap [28,44]. In May 2019, the study team hosted a community forum to share key learnings from the study to inform current practices and policies affecting TAY and other PEH, and to gather additional insights on the emergent thematic categories. Forum attendees were a diverse group of stakeholders, including TAY and other PEH, service providers, policymakers, and City/County public health officials (n ~ 100). The forum began with an exhibition of more than 20 photos and accompanying SHOWeD narratives. The academic and community co-leads then presented attendees with an overview of the research questions, methods, and findings. Post-presentation facilitated discussion focused on questions such as, “How do we ensure that available resources for TAY help them thrive rather than survive?” and elicited audience feedback on the findings presented. Discussion points from the forum, including attendee recommendations, further informed study findings.

## 3. Findings

Analyses yielded five overarching themes. Much of the findings related to the specific needs and experiences of TAY, however, many are relevant to diverse age groups of PEH.

### 3.1. The HAV Outbreak: Lack of Bathrooms and Long-Term Services and Resources to Meet TAY’s Needs

**Figure 1:**
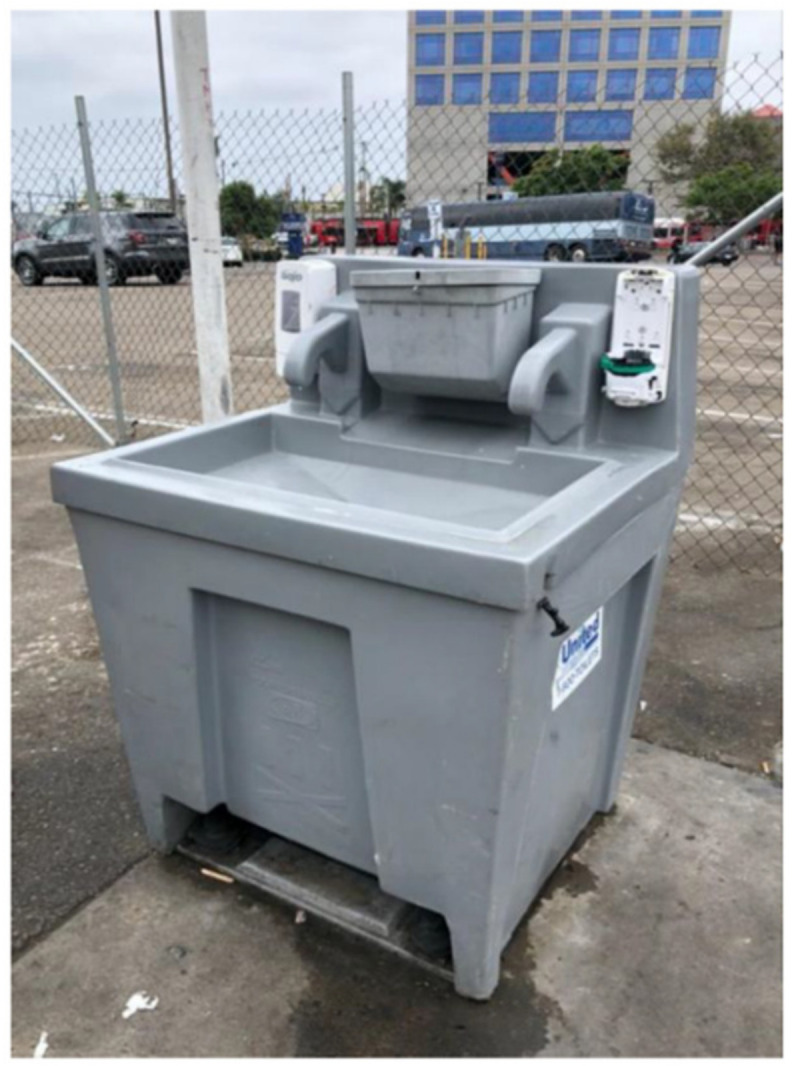
Location, Date: Downtown San Diego, 30/09/2018. Image Description: Image of a handwashing station on a public street; both dispensers are empty (one for hand soap, the other for hand sanitizer). “*[Handwashing stations] gives us a false sense of [safety]*”.—Quotation from SHOWeD Session; “*The County wouldn’t have cared [about the HAV outbreak] if it didn’t impact downtown or non-homeless people*”.—Quotation from member-checking session.

The multiple data sources suggested that TAY perceived public health efforts to address the HAV outbreak (e.g., handwashing stations, power-washing streets) as insufficient to address the root causes of the outbreak or to meet their basic needs. For example, in the SHOWeD sessions and member-checking sessions, TAY described HAV intervention efforts as temporary “Band-Aids” meant only to curb the spread of infection and appease housed residents and visitors rather than prevent future outbreaks. The asset map and photos/videos collected also supported this idea, demonstrating that the handwashing stations placed by public health authorities in key areas of San Diego with high proportions of PEH (e.g., downtown) were no longer in place shortly after the outbreak was declared over, despite PEH still needing access to handwashing facilities to maintain their health. Handwashing stations that remained often lacked soap and/or water, rendering them useless (See Figure 1).

**Figure 2:**
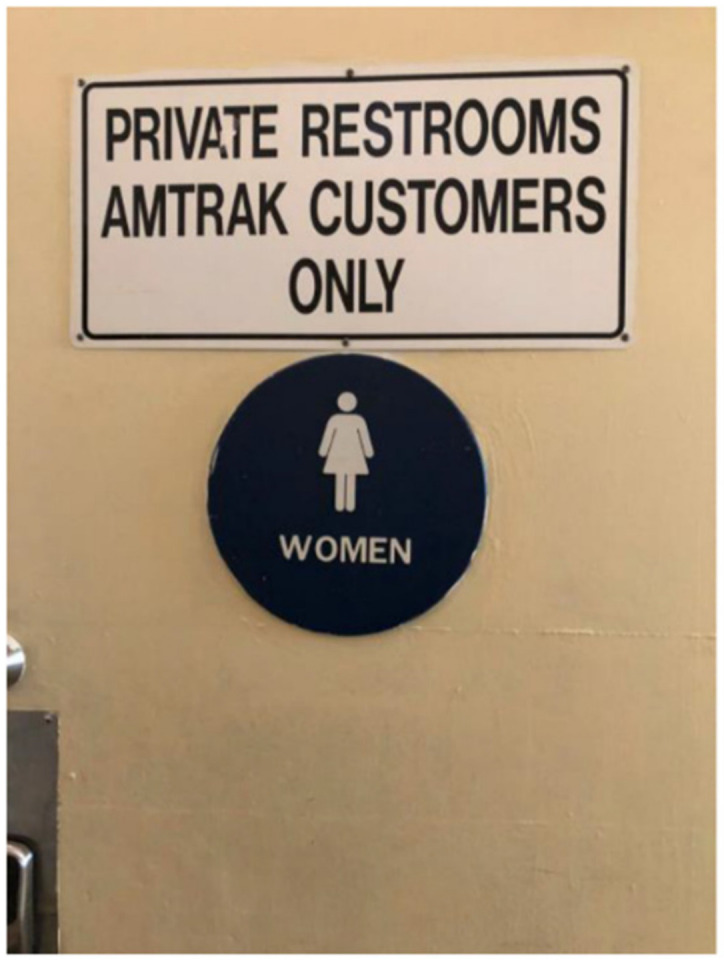
Location, Date: Downtown San Diego, 28/08/2018. Image Description: Close-up photo of a bathroom door at a train station; sign on door reads “Private Restrooms Amtrak Customers Only”. “*You can’t [use the bathroom] unless you have a [train] ticket*”.—Quotation from SHOWeD session.

**Figure 3:**
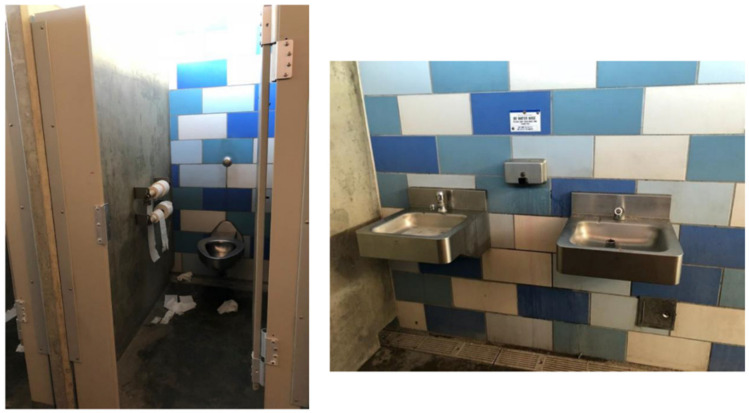
Location, Date: Beach neighborhood on south coast of San Diego, 21/08/2018. Image Description: Two photos of the women’s public bathroom—[Left] bathroom stall with toilet and toilet paper strewn on ground; [Right] two sinks with empty soap dispenser. “*That place should’ve been clean. At least, like inside that spot around that area should be clean for everybody to go to a clean spot instead of the dirtiness they always know*”.—Quotation from SHOWeD session.

Access to bathrooms emerged as a determinant of the HAV outbreak and health among TAY and other PEH in San Diego. For example, the photovoice data, asset map, early stakeholder interviews, and historical analysis demonstrated that there is a critical lack of public bathrooms in San Diego—something advocates and the San Diego Grand Jury have been raising to policymakers for several years [11,45]. In addition to the lack of bathrooms, the photovoice data suggested that available bathrooms were typically reserved for paying customers in local businesses (see Figure 2), or were unsanitary (trash accumulating in corners, dirty floors and toilets, empty soap dispensers). One of the community co-leads explained in a SHOWeD session analyzing the photos in Figure 3: “The more unclean you are, the more likely you are to get sick, or some kind of infection. When you’re on the streets, it’s so much harder to deal with [being sick] than if you’re at home. On the street, it’s like ‘okay, I got sick ‘cause I’m in a really dirty environment, the showers are closed by the time I get there, and now I’ve got Hepatitis A’”.

### 3.2. Stigma

**Figure 4:**
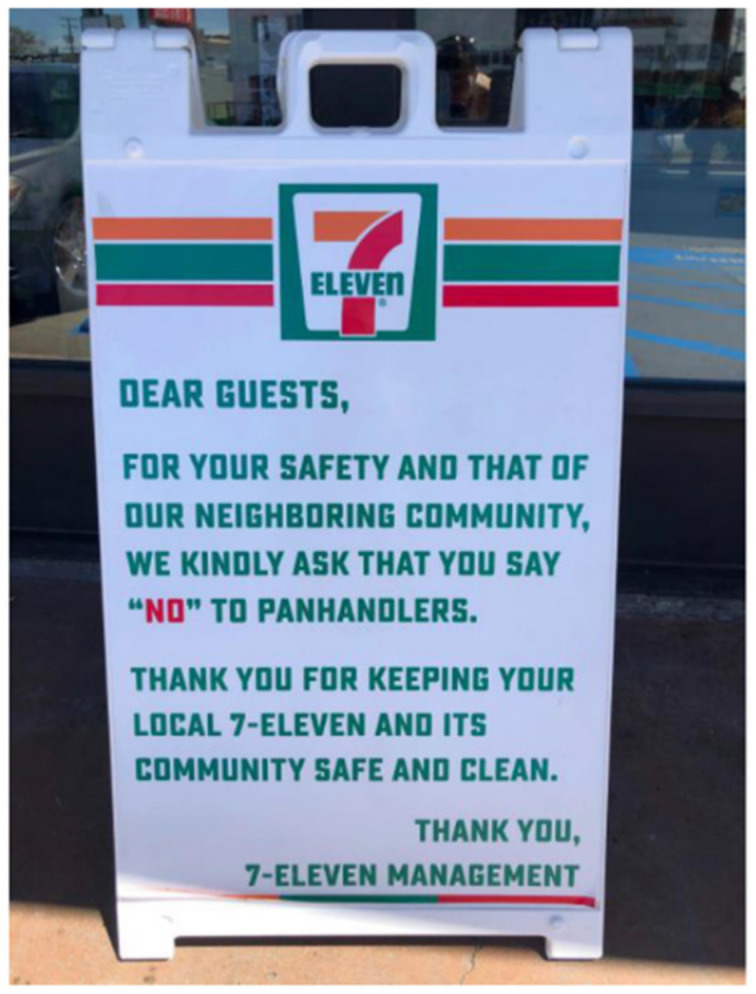
Location, Date: Central City of San Diego neighborhood, 07/04/2019. Image Description: A sign posted outside of the 7/11 convenience store that requests customers say “no to panhandlers” as a way to keep the store and local community “safe and clean”. “*This sounds like those signs that say ‘don’t feed the birds’*”.—Quotation from SHOWeD session.

The photovoice data in particular highlighted the stigmatization of homelessness as a key social and environmental determinant of health and well-being among TAY, echoing findings from past literature [13,46,47,48]. TAY participating in the community SHOWeD session and in the member-checking sessions also verbalized this point and suggested that stigma shaped how public health officials responded to the HAV outbreak. For example, many of the visual data points collected by the study team highlighted the various ways in which PEH and homelessness in general are stigmatized. For example, signs posted by local businesses used stigmatizing language about PEH (as demonstrated in Figure 4). SHOWeD analyses suggested that such language positions TAY and other PEH as a sub-human nuisance to the community who should not be offered help and support.

**Figure 5:**
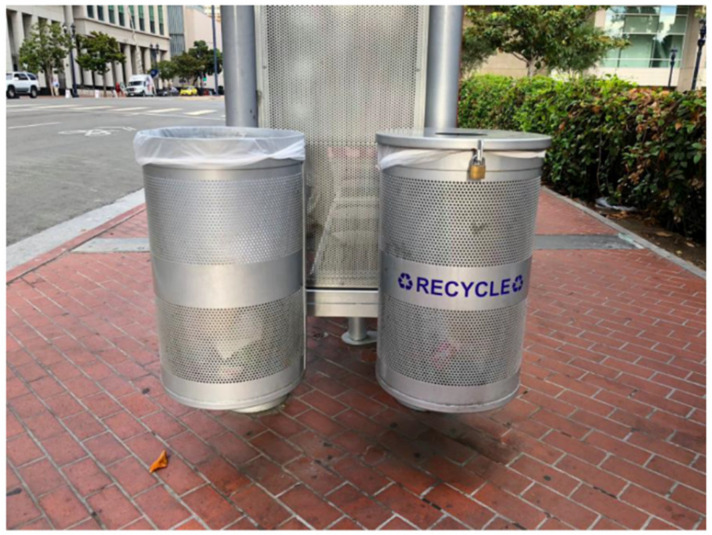
Location, Date: Downtown San Diego, 28/08/18. Image Description: Photo of two bins—an unlocked garbage bin next to a locked recycling bin. “*This is symbolic of who should be protected and how a community can be clean, by not promoting [or] helping those who are poor and/or homeless*”.—Quotation from SHOWeD session.

The study team documented several other visual symbols and features of the social and built environment which were stigmatizing to PEH. For example, Figure 5 depicts an unlocked trashcan next to a locked recycling bin in downtown San Diego. In the accompanying SHOWeD and member-checking sessions, this photograph was interpreted by TAY as symbolizing that items of no monetary value (i.e., trash) are freely available, but that items that are cast aside yet have monetary value (e.g., recyclable cans and bottles) must be protected or have their access restricted—presumably from PEH, who might generate resources outside of the formal economy through the sale or trade of such items [14].

Finally, stigma within programs and resources for TAY also emerged across the data sources, including stakeholder interviews, SHOWeD sessions, and member-checking sessions. For example, the criteria used to determine who will have priority access to housing-related resources was considered stigmatizing, with TAY who have a mental illness prioritized over those who do not [49]. The main tool used to determine a TAY’s service prioritization in San Diego is the TAY Vulnerability Index-Service Prioritization Decision Assistance Tool (TAY-VI-SPDAT)—a brief questionnaire administered by a service provider or other intake coordinator. Specific questions from the TAY-VI-SPDAT related to assessing mental health include:

- “Have you ever been diagnosed with a mental health condition?” (Yes/No/Refused/Don’t know); “If yes, what was your diagnosis?” (Anxiety/Bipolar/Depression/Schizophrenia/Other [Specify])
- “Have you ever been hospitalized for a psychiatric problem?” (Yes/No/Refused/Don’t know)
- “Do you ever see or hear things that other people don’t see or hear?” (Yes/No/Refused/Don’t know)” [50].

Using mental illness diagnoses as prioritization criterion in matching TAY to housing-related resources may further stigmatize and disadvantage TAY by associating homelessness with adverse mental health conditions and by creating additional barriers to accessing needed resources [51].

### 3.3. Resources Contain and Constrain

One powerful set of findings from the photovoice data (particularly the data collection prompt “a day in the life of a TAY accessing specific resources or services, or meeting basic needs”), and supported by asset mapping, was that many of the resources available to TAY and other PEH were geospatially located in a small area of downtown San Diego. This clustering of resources is likely a result of NIMBYism and associated community action against the provision of services for PEH in middle- and high-income neighborhoods outside of downtown [14,33,34,35]). Relatedly, many of the HAV interventions—e.g., handwashing stations—were also concentrated in and around the same downtown area.

Converging evidence across the photovoice data, asset map, and stakeholder interviews, exposed that there was a lack of coordination across housing-related and other social support resources. For example, complementary resources (e.g., showers and access to food) were provided at conflicting times, such that TAY were confronted with the difficult situation of having to choose between one resource or another rather than being able to access all essential resources. In SHOWeD and member-checking sessions, TAY noted delays in accessing resources or inability to access all resources because they had to wait in long lines, travel between buildings/agencies, and/or face denial of services due to lack of demonstration of sufficient need (e.g., documentation of a mental illness). These processes limited opportunities to travel to visit with friends or family, access or search for other resources (e.g., stable housing), or attend work and/or school. As one of the community co-leads explained during a SHOWeD session: “You would need a little more than [the available resources] for an actual day if you wanted to stay healthy and, like, do good”. Collectively, the data suggest that the clustering of services in a limited geographic area and the lack of coordination between resources serves to contain the movement of TAY and other PEH within specific parts of San Diego (i.e., downtown), as well as constrain their upward economic and social mobility.

**Figure 6:**
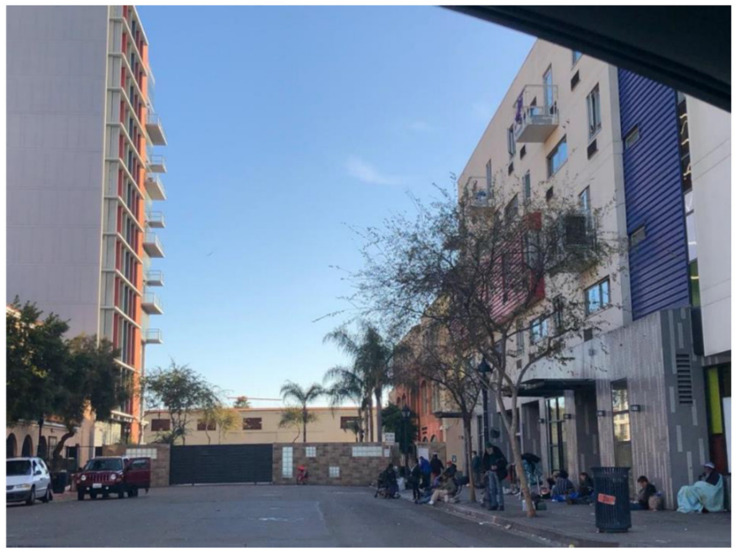
Date, Location: Downtown San Diego, 23/09/2018. Image Description: A wall separating the street view from the courtyard of a large service organization where TAY and other PEH access meals, showers, and other resources. “*There’s so many little things [the organization] could be doing, but literally all they have is the wall and then the resources [on the other side]. Like, they don’t care about anything else, as long as the [people] are hidden*”.—Quotation from SHOWeD session.

Finally, the SHOWeD and member-checking sessions suggested that the ways in which resources are distributed in San Diego serve to hide PEH from the housed public. For example, as depicted in Figure 6, PEH at one local community-based organization were literally separated from street view by a barrier which separated those seeking services on one side from “everyone else” on the other. Such a built environment feature intersects with the theme of stigma, suggesting that TAY and other PEH should be hidden rather than offered services in a non-stigmatizing, visible manner.

### 3.4. Criminalization and Over-Policing

**Figure 7:**
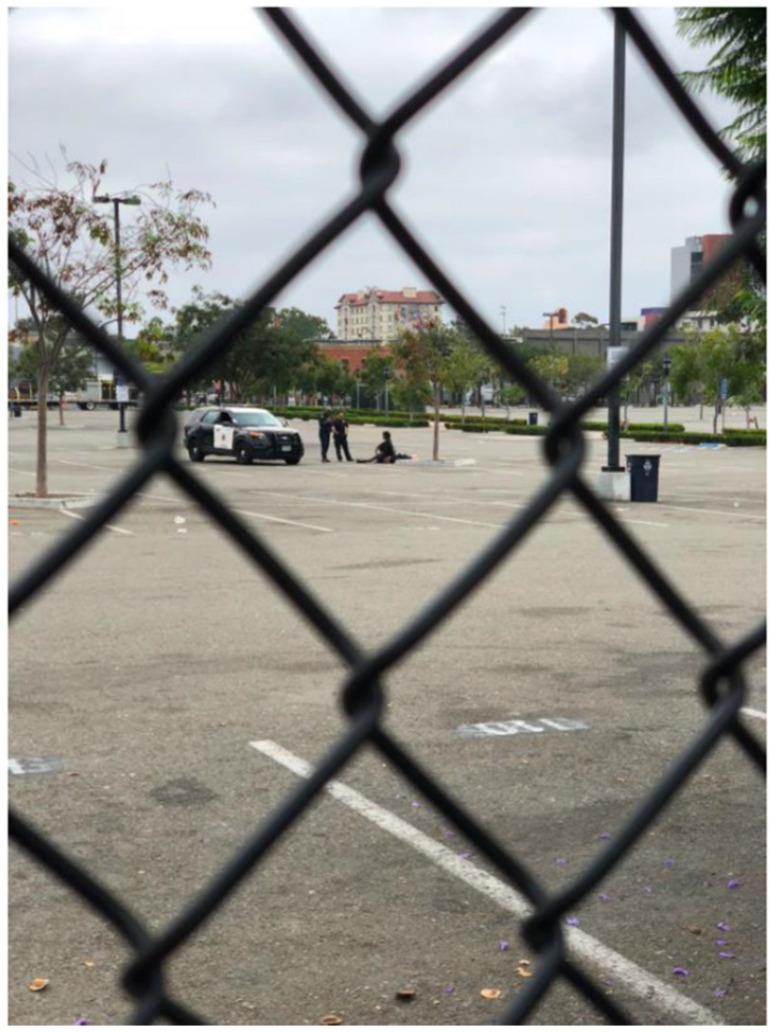
Location, Date: Downtown San Diego, 09/29/2018. Image Description: Photo taken through a chain link fence of two police officers outside of their car standing over an individual who is sitting on the ground in an empty parking lot. “*If [there were] resources to help the people, police [wouldn’t] need to be patrolling*”.—Quotation from SHOWeD session. “*The laws are smoke and mirrors*”; “*The way the system is set up, it gives cops immunity and they abuse it*”.— Quotations from member-checking session.

The study team regularly witnessed the criminalization and over-policing of TAY and other PEH, particularly in downtown, a phenomenon documented in the existing literature on homelessness [14,34,35,52]. For example, during study team meetings at the downtown public library where many PEH seek shelter or access resources (e.g., charging phones, using bathrooms, accessing Internet, reading books), police cars were frequently circling the library surveilling groups of people who appeared to be experiencing homelessness, and/or asking them to leave the area (see Figure 7).

The SHOWeD sessions and member-checking sessions suggested that TAY perceived this as constant policing of their movements simply because they were unhoused. Some TAY in the member-checking sessions explained that people of color experiencing homelessness were even more heavily policed than those who are white: “Don’t be a person of color and homeless, because now they think there’s drugs involved”. In addition, TAY in the member-checking sessions discussed how the HAV outbreak presented a new opportunity to police TAY and other PEH: “Let’s give [PEH] handwashing stations, power-wash the streets, and then send the police to clean them up. This is what the mayor did [during the outbreak]”.

In several SHOWeD sessions, the study team discussed signage of multiple ordinances in San Diego which were interpreted by the community co-leads as functioning solely to criminalize PEH. For example, the study team collected photos of signs which forbade “loitering” in public spaces, suggesting that PEH cannot sit or lay down in a public area without fear of being harassed and/or ticketed by police. At the community forum, this theme emerged again when a participant working security for businesses in the downtown area explained that much of their job involved removing PEH from the front of buildings. The participant lamented this function of their job and explained that such actions create divisions between PEH and security guards who wish to help rather than stigmatize and criminalize them.

For several years, the San Diego Police Department has partnered with psychiatric and mental health clinicians to decrease the criminalization of homelessness and meet the specific needs of PEH through programs such as the Homeless Outreach Team (HOT). The study team learned from the stakeholder interviews that the HOT is often called when organizations (e.g., local libraries) identify a person experiencing homelessness with an urgent need, such as emergency mental health care. Though members of the study team witnessed police patrolling downtown nearly every instance they visited (often several times per month), the study team never observed any HOT-marked vehicles or individuals clearly identifiable as members of the HOT in downtown (or elsewhere in San Diego). In addition, neither the community co-leads nor TAY participating in the community SHOWeD or member-checking sessions indicated having any personal experiences with the HOT.

### 3.5. Need for More Resources with Fewer Restrictions

**Figure 8:**
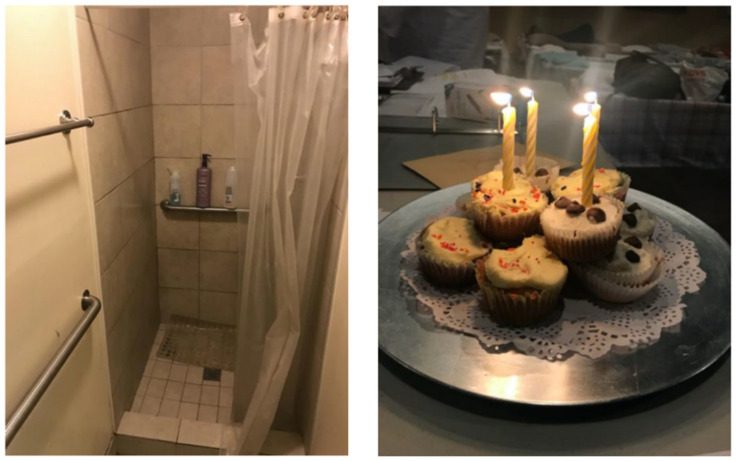
Location, Date: Emergency overnight shelter for TAY in central City of San Diego neighborhood, 21/08/2018. Image Description: [left] Photo of a shower for shelter guests; [right] photo of cupcakes with lit candles to celebrate a shelter guest’s birthday. “*[This] overnight [shelter is] where the youth can be free and not worry about the dangers of the outside world*”.; “*[The shelter] is a pro and a con. You get a shower, food, clothing, but you only get [it for] a short time*”.—Quotations from SHOWeD session.

**Figure 9:**
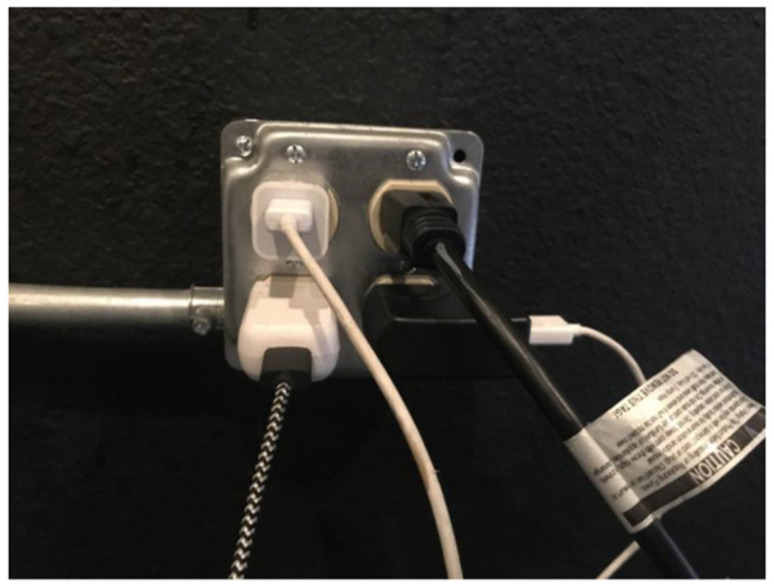
Location, Date: Emergency overnight shelter for TAY in central City of San Diego neighborhood, 21/08/2018. Photo Description: Photo of a power outlet with multiple power cords charging phones and other electronics. “*Phones are so critical to get needs met, connecting to others. How often can TAY get access to power*?”—Quotation from SHOWeD session.

Because TAY and other young PEH have specialized needs given their age and developmental stage, there are youth- and TAY-specific resources available in San Diego, such as drop-in programs, emergency shelters, and short- and long-term housing programs. However, each of these services is extremely limited in terms of who may access them, when, where, and for how long. For example, one of the few available emergency shelters for youth and/or TAY—located in a central City of San Diego neighborhood—operated only one night a week (and since the conclusion of this study, has been shut down). The study team learned that this resource was one of the few places where TAY could access showers, food, a place to sleep, and charge their phones, which had no restrictions other than age. As depicted in Figure 8 and Figure 9, these limited resources are critical for TAY and provide, albeit limited, a semblance of “normalcy”.

As discussed earlier, many of the available programs for TAY have restrictions or requirements for participation that may further stigmatize TAY and constrain opportunities for well-being (e.g., prioritizing housing-related resources for those who have a documented mental illness). Requirements of this kind suggest that simply being unhoused is insufficient to warrant a young person worthy of support. Because of these requirements, some TAY may forgo participating in housing programs altogether. In one of the member-checking sessions, a participant explained that TAY accessing housing-related resources are often expected to “play up” or continuously share any challenges they are dealing with (e.g., mental illness), such that they become a “dog and pony show” for the organizations serving them.

In addition, several TAY of color participating in the community SHOWeD session and the member-checking sessions indicated that they experienced individually mediated racism and other forms of discrimination by staff within certain TAY-serving housing programs, negatively impacting their experience within the programs and limiting their opportunities during and after program participation. For example, a TAY from one of the member-checking sessions explained: “You can’t be young, Black, and trans[gender] in this city”. Such testimonials highlight how intersectional oppression can further exacerbate the quality of experience with resources for TAY, or lead to the exclusion of some TAY from needed resources.

## 4. Discussion

By utilizing diverse qualitative methodologies—principally photovoice—this CBPR study examined how stigma and the social and built environment shapes health among TAY, how these environments may have contributed to the 2016-18 HAV outbreak in San Diego, and TAY’s perceptions of HAV-related interventions. The CBPR approach allowed the study team to leverage the diverse insights and strengths of both the community and academic partners to critically examine an issue of local importance. Results suggested that the stigmatization of TAY and other PEH may have been a key factor that both guided and was perpetuated by the public health response to the outbreak.

Across the various data sources, the stigma of homelessness pervaded the language used to describe the HAV outbreak and the attribution of blame for the outbreak. Such framing contributed to an “us” (i.e., housed and to be protected) vs. “them” (i.e., unhoused and infected) social discourse. Indeed, the temporary nature of public health HAV interventions was perceived by the study team, TAY participating in the community SHOWeD session and the member-checking sessions, and attendees at the community forum as an enactment of stigma. These interventions were considered “Band-Aids” that demonstrated public health authorities’ predilection towards minimizing sustained contact with PEH, or lack of investment in long-term solutions to homelessness. Furthermore, the multiple data sources provided evidence of the diverse ways that stigma against TAY and other PEH is built into the landscape of San Diego. For example, stigma was evident in the signage used to refer to PEH and control their access to resources and space throughout San Diego (e.g., signs discouraging the support of panhandlers to protect the health and safety of the community). Analysis of photographs of signage were consistent with the experiences reported by TAY participating in community SHOWeD and member-checking sessions and the community forum, in which they described the act of merely existing as a TAY or other PEH in a public space as being criminalized. This well-documented phenomenon [14,34,35,52] was perceived as further marginalizing a group that already lacks power and control over personal space by creating barriers to opportunities for health and social equity. Additionally, the HAV interventions (e.g., handwashing stations) and general resources for TAY and other PEH were physically located in a restricted geographic region, with brick and mortar resources sometimes hidden from the view of the (housed) public. Taken together, the findings indicated that stigma in the provision of and access to resources create systemic barriers to health among TAY and other PEH in general, and perhaps contributed to their increased risk for HAV and the attribution of responsibility they received for the HAV outbreak.

Although the study focused on a local issue, the findings align with prior observational and intervention research on factors contributing to the chronic cycle of homelessness, especially how inefficiencies and lack of coordination of homelessness services and over-policing can limit healthcare access and treatment, curb the attainment of long-term housing, and further stigmatize and constrain economic and social mobility among PEH [52,53,54,55]. In addition, the findings support previous theory and research on stigma [15,16,17,18] and the stigmatization of economically marginalized TAY and PEH [13,47,56,57]. For example, prior research finds that the mere attribution of the label “homeless” to individuals often conjures associations of socially undesirable attributes and qualities (e.g., physical, mental, and behavioral) [13,58]. In this study, HAV appeared to become one of the undesirable attributes or qualities ascribed to homelessness. Related to Link and Phelan’s Stigma Power concept [17], the findings emphasize that the social discourse and public health response related to HAV in San Diego between 2016-2018 served to keep TAY and other PEH down (e.g., in the position of blame for the HAV outbreak; the “us vs. them” discourse; criminalization and over-policing of PEH), and to keep them away (e.g., restricting resources geographically and offering them on a time-limited basis; not including the voices of PEH in local media coverage or the input of PEH planning solutions for the HAV outbreak).

The current study brought into sharp focus the additional burden that TAY face given that housing-related resources are substantially limited and often reserved for those deemed the worthiest of help (e.g., those who have a documented mental illness). Ultimately, however, the data collected and iterative inquiry uncovered processes of social and environmental health inequity that were cross-cutting for diverse groups of PEH rather than specific to TAY (e.g., stigma; criminalization and over-policing; containment and constraining effects of resources). Findings also support past research on homelessness, criminalization, and the ways in which structural barriers and stigma hinder PEH’s opportunities to access critical resources and secure long-term housing [34,35,51,59].

By examining the HAV outbreak using diverse, iterative qualitative methods, this study exposed social and environmental determinants that may have both exacerbated the spread of an infectious disease in a vulnerable population, and increased risk for the chronic cycle of future homelessness among TAY. The methods also unveiled how local public health efforts to curb the outbreak may have contributed to health inequity by failing to consider and address upstream factors that contribute to acute and chronic disease risk among TAY and other PEH. It is important to note that focusing on infectious disease containment in the face of an outbreak is imperative [60]; however, the current study—which was co-led by community members of a primary affected population—suggested that inadequate focus on social and environmental determinants of health may have perpetuated stigma. Indeed, prior research has demonstrated that the stigmatization of homelessness could lead those with greater power (e.g., policymakers, public health authorities, medical professionals, researchers, the housed) to view homelessness and any issues related to homelessness (e.g., greater susceptibility to infectious disease, engagement in survival economy) to be issues that result from personal fault (e.g., poor decision-making) rather than systemic inequity (e.g., oppression, gaps in social safety net, inadequacies in health care coverage) [13]. By focusing narrowly on HAV from an infectious disease intervention lens rather than a health equity and social determinants of health lens, public health officials may have inadvertently caused additional harm to TAY and other PEH because they did not adequately address the multi-level, complex factors shaping health among this diverse population. For example, consistent with the sentiments of the participants that most HAV interventions were “Band-Aids”, the handwashing stations, porta-potties, and power-washing streets were temporary remedies for the ongoing, systemic issue of a dearth of freely accessible, clean, and well-stocked public bathrooms to sustain the health of the entire public (i.e., unhoused and housed).

### 4.1. Study Limitations

The findings of the current study must be interpreted considering its limitations, which future studies may address. There are multiple ethical considerations in photovoice methodology (e.g., respect for persons; safety of researchers and safety and privacy of anyone in a photographic view; consent, etc.) [61,62] that precluded some subject matter and topics from being photographed. Thus, some data points in the analysis were captured predominantly via supporting methods, e.g., discussions about criminalization and over-policing during SHOWeD and member-checking sessions. Furthermore, photovoice relies heavily on phenomena that can be captured visually. Thus, some of the themes found in the study (e.g., intersectional oppression, such as racism experienced within service settings) were difficult to capture in images although they came up in multiple discussions. In addition, while the study team was unable to cover the entire San Diego region during photovoice data collection, they were confident that they achieved saturation in terms of data collected and themes identified, particularly upon member-checking and discussion at the community forum. The research was led by a small study team, with only two community partners who completed participation throughout the entire duration of the project (compared to the relatively larger number of academic partners, which included the academic co-leads and student research assistants). Although it was always the case that the community co-leads were addressed and treated as partners, paid throughout the study, and included as authors in this article, it is important to acknowledge the inherent power and resource dynamics in the research setting that could have shaped decision-making throughout the study. In addition, the study team could have included other TAY from outside of the academic and community partner co-led team in the collection of photos, and gathered insights from other stakeholders (e.g., law enforcement personnel and policymakers with direct experience with TAY and other PEH). The inclusion of such additional perspectives may have further informed the scope of the research questions, data collection efforts, and study findings. Similarly, because the team was co-led by and predominantly focused on the experiences and perceptions of TAY, it is possible that different areas of inquiry or perspectives could have been explored had the study team included other communities of PEH (e.g., mothers experiencing homelessness). In addition, the study team’s exclusive use of qualitative methods limit the extent to which the findings are generalizable to future HAV or other infectious outbreaks, and/or for diverse communities of PEH. These findings are, however, highly transferable [63,64] to myriad other urban areas with high rates of PEH, and can inform approaches to providing sustainable and responsive services to TAY and other PEH (e.g., regarding enhanced coordination of resources, sanitation, and housing), which may prevent infectious disease outbreaks. Finally, while the community forum was a critical element of the study such that emergent findings could be validated and information disseminated to relevant audiences, the study team cannot yet confirm the extent to which information shared has led to concrete changes (e.g., in policy) that support health and well-being among TAY and other PEH. As knowledge dissemination and advocacy are key elements of effective CBPR processes, future research is needed to determine the influence of this study’s findings on relevant practice and policy.

### 4.2. Study Implications

The findings have several implications for research on infectious disease outbreaks affecting PEH broadly, and research and public health practice with this population in general. Given the overwhelming degree of evidence indicating the role that stigma played in framing the narrative and public health response to HAV in San Diego and homelessness in general, the findings indicate the critical need for research, public health prevention and intervention efforts, and policy development to adopt explicitly anti-oppressive and community-engaged practices. At minimum, involvement and input from PEH in the development and implementation of research, public health practices, and processes related to policymaking could ensure that populations affected by and targeted for intervention trust public health authorities and the academic establishment, and that research programs, policies, and public health interventions are responsive, sustainable, and culturally and communally grounded [65]. As described by the findings, the apparent lack of input from PEH in planning public health efforts in the wake of the HAV outbreak resulted in a disconnect between the public health response to HAV and the lived experiences of TAY and other PEH. As such, TAY and other PEH were positioned as non-community member “others” responsible for the outbreak who received interventions that did not sufficiently meet their acute and long-term needs. In addition, because the photovoice analysis highlighted that upstream determinants of health played critical roles in both the transmission of infectious disease and the perpetuation of conditions that drive health inequities, the results strongly suggest the need for researchers, public health authorities, and policymakers to consider the social determinants of infectious disease outbreaks in planning interventions, rather than narrowly focusing on disease containment.

The data in the current study were particularly relevant in demonstrating the power of language and representation in framing the outbreak. Signage, the language of public health messaging and media coverage, and even the prevailing terminology used to describe PEH (i.e., the stigma-inducing “homeless people”) were all perceived by TAY and other PEH to exacerbate or perpetuate health inequities during the outbreak. Future efforts to address these phenomena include responsible media practices that encourage the inclusion of perspectives of PEH in any stories or content that focus on their experiences, the use of person-first language (e.g., “people experiencing homelessness” rather than “homeless people”) to reinforce that homelessness is not a permanent trait, and efforts by media, researchers, and the public to cultivate a deeper understanding of the diverse personal narratives of PEH. Participatory research leveraging storytelling and oral histories as novel, in-depth qualitative methods [66,67] may also help to dismantle attitudes of stigma of homelessness by showcasing not only the myriad ways that individuals can become homeless and experience homelessness, but also the many (unheard) stories of strength and resilience.

As the current COVID-19 pandemic has demonstrated, HAV is not the only large-scale infectious disease outbreak in which patterns of transmission and access to health interventions (e.g., ability to engage in social distancing; ability to work while under self-quarantine; access to regular handwashing) intersect with power, class dynamics, and access to safe space. In the U.S., disparities in COVID-19 infections and complications have been especially pronounced among stigmatized and marginalized groups, including PEH [68,69]. As this evidence continues to emerge, it is even more clear that the common political and public health rallying cry of “we are all in this together” does not apply to groups marginalized by structural inequity [70]. As such, local and national governments must ensure that equitable prevention and infection control strategies are implemented in the wake of infectious disease outbreaks such that all citizens—regardless of social location—are afforded responsive approaches to prevention resources, healthcare, and social support services.

## 5. Conclusions

The purpose of this CBPR photovoice study was to understand—from the perspective of TAY—how stigma and the social and built environment shapes health among TAY in San Diego, how these environments may have contributed to the 2016–18 HAV outbreak, and TAY’s perceptions of HAV interventions. Overall, the findings suggest that the social enactment of stigma (in language and public health practices), the physical representation of stigma in the built environment, and lack of power experienced by TAY and other PEH all served to position PEH as a high-risk group for HAV and an easy scapegoat for the outbreak. In addition, the findings highlight the need for more resources with fewer restrictions that are better coordinated and that support rather than constrain opportunities for economic and social mobility among TAY and other PEH. Such changes are critical for preventing future infectious disease outbreaks predominately affecting such “high-risk” groups and for promoting heath equity among TAY and other PEH in San Diego.

## Figures and Tables

**Table 1:** Data collection and validation overview and study timeline.

Method	Sampling Frame	Goal of Method	Sample Size	Data Collectors	Oct.–Dec. ’17	Jan.–Mar. ’18	Apr.–Jun. ’18	Jul.–Sept. ’18	Oct.–Dec. ’18	Jan.–Mar. ’19	Apr.–May ’19
Primary Method: Photovoice (Three Components)											
Photography and Videography	Two beach neighborhoods on the South and central coast of San Diego, two neighborhoods in downtown San Diego, a central City of San Diego neighborhood	To use photography and videography to document how the social and built environment shapes health among TAY, how these environments may have contributed to the HAV outbreak, and TAY’s perceptions of HAV interventions	5 neighborhoods, 250+ photos/videos	Study team (i.e., academic and community co-leads, student research assistants)				X	X	X	X
SHOWeD Sessions	Study team	To use narratives and critical discussion to examine perceived meanings behind photos and videos collected	12 SHOWeD sessions	Study team				X	X	X	X
Community SHOWeD Session	TAY	To collect additional community insight on existing photovoice data	7 TAY participants	Study team						X	
Supporting Methods											
Historical Analysis	News/Media articles dated 24/05/2017–15/01/2019	To understand how HAV and homelessness was portrayed to the public via the media	30 articles	Academic co-leads and research assistants	X	X	X	X	X	X	
Asset Mapping	Resources for HAV outbreak advertised by County health and human services; resources for TAY experiencing homelessness in San Diego	To understand how HAV interventions (e.g., handwashing stations) and resources for TAY were geospatially distributed	N/A	Study team				X	X	X	
Validation											
Stakeholder Interviews	Individuals working with or volunteering with organizations serving PEH; individuals working for the City/County of San Diego; public health officials	To develop project scope, identify extent to which findings resonate with key informants, and contextualize results and their implications	10 stakeholders	Academic co-leads and research assistants	X				X	X	
Member- Checking Session (1)	TAY serving on a youth advisory board to address homelessness in San Diego	To assess whether findings from SHOWeD sessions and analysis of supporting methods reflected the experiences of TAY who were not involved with the data collection process and who have perspectives and experiences that may differ from members of the study team	4 TAY participants	Study team					X		
Member-Checking Session (2)	TAY participating in a housing program for LGBTQ youth and program staff	To assess whether findings from SHOWeD sessions and analysis of supporting methods reflected the experiences of diverse TAY who were not involved with the data collection process and who have perspectives and experiences that may differ from members of the study team	6 TAY participants, 1 program staff member	Study team						X	
